# Endoplasmic reticulum stress induces *PRNP *prion protein gene expression in breast cancer

**DOI:** 10.1186/bcr3398

**Published:** 2013-03-12

**Authors:** Marc-André Déry, Julie Jodoin, Josie Ursini-Siegel, Olga Aleynikova, Cristiano Ferrario, Saima Hassan, Mark Basik, Andréa C LeBlanc

**Affiliations:** 1 Bloomfield Center for Research in Aging, Lady Davis Institute for Medical Research, Sir Mortimer B. Davis Jewish General Hospital, 3755 ch. Côte Ste-Catherine, Montréal, QC H3T 1E2, Canada; 2 Department of Neurology and Neurosurgery, McGill University, 3801 University Street, Montréal, QC H3A 2B4, Canada; 3 Segal Centre for Cancer Research, Lady Davis Institute for Medical Research, Sir Mortimer B. Davis Jewish General Hospital, 3755 ch. Côte Ste-Catherine, Montréal, QC H3T 1E2, Canada

## Abstract

**Introduction:**

High prion protein (PrP) levels are associated with breast, colon and gastric cancer resistance to treatment and with a poor prognosis for the patients. However, little is known about the underlying molecular mechanism(s) regulating human PrP gene (*PRNP*) expression in cancers. Because endoplasmic reticulum (ER) stress is associated with solid tumors, we investigated a possible regulation of *PRNP *gene expression by ER stress.

**Methods:**

Published microarray databases of breast cancer tissues and breast carcinoma cell lines were analyzed for PrP mRNA and ER stress marker immunoglobulin heavy chain binding protein (BiP) levels. Breast cancer tissue microarrays (TMA) were immunostained for BiP and PrP. Breast carcinoma MCF-7, MDA-MB-231, HS578T and HCC1500 cells were treated with three different ER stressors - Brefeldin A, Tunicamycin, Thapsigargin - and levels of PrP mRNA or protein assessed by RT-PCR and Western blot analyses. A human *PRNP *promoter-luciferase reporter was used to assess transcriptional activation by ER stressors. Site-directed mutagenesis identified the ER stress response elements (ERSE). Chromatin immunoprecipitation (ChIP) analyses were done to identify the ER stress-mediated transcriptional regulators. The role of cleaved activating transcription factor 6α (ΔATF6α) and spliced X-box protein-1 (sXBP1) in *PRNP *gene expression was assessed with over-expression or silencing techniques. The role of PrP protection against ER stress was assessed with PrP siRNA and by using *Prnp *null cell lines.

**Results:**

We find that mRNA levels of BiP correlated with PrP transcript levels in breast cancer tissues and breast carcinoma cell lines. *PrP *mRNA levels were enriched in the basal subtype and were associated with poor prognosis in breast cancer patients. Higher PrP and BiP levels correlated with increasing tumor grade in TMA. ER stress was a positive regulator of *PRNP *gene transcription in MCF-7 cells and luciferase reporter assays identified one ER stress response element (ERSE) conserved among primates and rodents and three primate-specific ERSEs that regulated *PRNP *gene expression. Among the various transactivators of the ER stress-regulated unfolded protein response (UPR), ATF6α and XBP1 transactivated *PRNP *gene expression, but the ability of these varied in different cell types. Functionally, PrP delayed ER stress-induced cell death.

**Conclusions:**

These results establish *PRNP *as a novel ER stress-regulated gene that could increase survival in breast cancers.

## Introduction

Growing evidence indicates that prion protein (PrP) is associated with cellular survival. PrP confers neuroprotection against serum-deprivation [1], Bax protein [2-7], oxidative stress [8], ischemia [9] and PrP mutants or prion-like protein Doppel [10,11]. The anti-cell death activity of PrP also occurs in peripheral cell types and has been associated with several types of cancer. High levels of PrP induce resistance of MCF-7 breast carcinoma cells to tumor necrosis factor alpha, Tumor Necrosis Factor-Alpha-Related Apoptosis-Inducing Ligand (TRAIL)-, and Bax-mediated apoptosis [4,12,13]. In estrogen receptor negative breast tumors, PrP expression is associated with chemotherapy resistance [14]. Non-glycosylated and unprocessed non-glycophosphatidyl inositol anchored PrP is expressed in human pancreatic ductal adenocarcinoma and melanoma cell lines and confers increased proliferation, invasiveness and growth to these cells by interacting with the actin-regulating filamin A protein [15,16]. PrP is also over-expressed in gastric cancers [17], associated with resistance to chemotherapy and poor prognosis [18], and promotes proliferation, invasion and metastasis [19,20]. The induction of PrP in gastric cancer is related to hypoxia [21], and PrP is associated with increased activation of Akt, increased levels of Bcl-2, decreased levels of Bax and a dysregulation of calcium-related genes [22,23]. PrP is also implicated in colon cancer. PrP levels are higher in the most aggressive colon cancer cell lines [24] and in higher-grade human colorectal carcinomas [25]. Antibodies against PrP decrease cellular proliferation of colon carcinoma HCT116 cells and also decrease xenograft tumor growth in combination with irinotecan chemotherapy [24]. Furthermore, *PRNP *gene expression correlates with colorectal cancer recurrence [26].

Preliminary evidence suggests that endoplasmic reticulum (ER) stress may also regulate *PRNP *gene expression in breast cancer cells. Brefeldin A (BFA) significantly increases PrP levels in breast carcinoma MCF-7 cells [27]. Two other ER stress-inducing compounds, bisphenol A or misfolded surfactant protein C, increase PrP levels in mouse testicular Sertoli TTE3 and human embryonic kidney 293 (HEK293) cells [28,29]. ER stress initially induces a survival response via the unfolded protein response (UPR); however, prolonged activation leads to apoptosis [30]. The UPR triggers protein refolding [31], attenuation of protein translation [32] and degradation of misfolded proteins [33] via three transmembrane proteins; the activating transcription factor 6α (ATF6α), the double-stranded RNA-activated protein kinase-like ER kinase (PERK) and the inositol-requiring enzyme 1α (IRE1α). Under ER stress, ATF6α translocates to the Golgi apparatus and is processed by site-1 and site-2 proteases to generate cytosolic cleaved ATF6α (ΔATF6α). ΔATF6α translocates to the nucleus and activates transcription of ER chaperones, XBP1 and the CCAAT/enhancer-binding protein homologous protein (CHOP) [34,35]. IRE1α cleaves the mRNA of XBP1 generating a spliced variant (sXBP1) that acts as a transcriptional activator of chaperones and genes involved in protein degradation [36]. PERK phosphorylates the eukaryotic initiation factor 2α (eIF2α) and leads to a general decrease in protein translation. Phosphorylation of eIF2α also causes a preferential translation of the activating transcription factor 4 (ATF4) that induces genes mainly involved in amino acid metabolism and stress response [37]. ΔATF6α and sXBP1 recognize an ERSE defined in glucose-regulated genes by a consensus CCAAT-N9-CCACG sequence [35]. In addition, an ATTGG-N-CCACG ERSE-II motif also responds to ER stress [38]. The ERSE motifs can accommodate nucleotide substitutions and operate in a bi-directional manner [35,38-43].

Given that ER stress-mediated activation of the UPR occurs in cancer tissues and cell lines [44,45], we investigated its effect on *PRNP *gene expression in breast cancer. We found that high mRNA levels of the ER stress marker, immunoglobulin heavy chain binding protein (BiP), correlated with high levels of PrP mRNA in breast cancer tissues and cell lines, both at the RNA and protein level. The high PrP mRNA levels were associated with a poorer prognosis and with the acquisition of a basal phenotype, which is associated with poor patient survival. We then explored the underlying molecular pathway by which ER stress increased *PRNP *gene expression in the breast carcinoma MCF-7 cell line. Our results showed that ER stress increased endogenous PrP levels through ERSE motifs of the human *PRNP *promoter. Among the transactivators induced by the three UPR pathways, ATF6α and sXBP1 were both capable of transactivating *PRNP *gene expression. Silencing PrP exacerbated ER stress-induced apoptosis in the MCF-7 and MDA-MB-231 cell lines. These results establish *PRNP *as a novel ER stress-regulated gene and implicate PrP as a pro-survival factor in breast cancer cell lines.

## Material and methods

### Tissue microarrays and immunohistochemistry

All human tissue used for research was obtained by ethical consent of patients [46] and protocol was approved from the Centre Hospitalier de l'Université de Montréal Research Ethics Committee under Canadian Institutes of Health Research (CIHR) guidance rules and in compliance with the Helsinki Declaration. Tissue microarrays (TMAs) were composed of formalin-fixed, paraffin-embedded breast cancer biopsies collected at the Centre Hospitalier de l'Université de Montréal. After re-hydration of the TMA, antigen retrieval was performed by heating the slides in Antigen Unmasking Solution (Vector Laboratories, Burlingame, CA, USA) with a steam oven. Immunohistochemical staining was performed on a Dako Autostainer Plus (Dako, Burlington, ON, Canada). Briefly, Peroxidase Block solution and Protein Block solution was applied on slides before the anti-PrP (1:500, 3F4) or anti-BiP (1:500, Cell Signaling Technologies, Danvers, MA, USA, #3177) antibodies. The 3F4 epitope-spanning peptide PrP_106-114 _(Anaspec, Fremont, CA, USA) or BiP blocking peptide (Cell Signaling) were used to adsorb their respective antibody immunoreactivity. Slides were developed with the Envision Flex DAB Chromagen system (Dako, Burlington, ON, Canada) and counterstained with hematoxylin. Slides were mounted in permount and scanned with a Mirax Scan 150 BF/FL Digitizer (Carl Zeiss, Toronto, ON, Canada).

### Cell culture and treatments

MCF-7 cells (ATCC, Manassas, VA, USA) were seeded at 1 × 10^6 ^cells per 35 mm well and treated with 5 μg/mL BFA, 3.25 μg/mL Thps, 2.5 μg/mL TM or their vehicle (Biomol Research Laboratories, Plymouth Meeting, PA, USA) in complete cell culture media and in the absence or presence of 1 μg/mL actinomycin D or 20 μg/mL cycloheximide (Sigma-Aldrich, Oakville, ON, Canada) for the indicated times. Serum deprivation of MCF-7 cells was done for 6 and 18 hours. Basal cell lines MDA-MB-231 (ATCC) and HS578T (ATCC) were seeded at 0.8 × 10^6 ^per 35 mm well and treated for 20 h with 10 μM 4-Phenyl-Butyric Acid (4-PBA). HCC1500 cells were obtained from ATCC and were cultured at 1 × 10^6 ^cells per 35 mm well in RPMI and 10% FBS. MDA-MB-231, HS578T and HCC1500 cells were all treated with 5 μg/mL of BFA, Thps or TM.

### Protein extraction and western blot analyses

Cells were lysed on ice in lysis buffer (150 mM NaCl, 2 mM Ethylenediaminetetraacetic acid (EDTA), 0.5% Triton X-100, 0.5% sodium deoxycholate, 50 mM Tris-HCl, pH 7.5, 38 μg/mL 4-(2-Aminoethyl) benzenesulfonyl fluoride (AEBSF), 0.5 μg/mL leupeptin, 0.1 μg/mL pepstatin, 0.1 μg/mL N-a-p-tosyl-L-lysine chloromethyl ketone hydrochloride, 4 mM sodium orthovanadate, 20 mM sodium fluoride). Protein concentration was determined with the BCA Protein Assay Reagents (Fisher Scientific, Toronto, ON, Canada). Proteins were precipitated in methanol, dried and solubilized in Laemmli sample buffer before being boiled, migrated on SDS-PAGE gel and transferred to PVDF membranes. Membranes were blotted for PrP (3F4 1:2,000), β-actin (AC-15 1:5,000, Sigma), CHOP (B-3 1:1,000, Santa Cruz Biotechnology, Santa Cruz, CA, USA), BiP (H-129 1:250, Santa Cruz Biotechnology), ATF6α (1:500, Imgenex, San Diego, CA, USA), eIF2α (1:500, Cell Signaling), phosphorylated eIF2α (peIF2αSer51 1:500, Cell Signaling), XBP1 (M-186 1:200, Santa Cruz Biotechnology), Bax (2D2 or N-20 1:2,000 or 1:3,000, Sigma), Bim (Y-36 1:5,000, Epitomics, Burlingame, CA, USA) and Bcl-2 (100 1:250, Santa Cruz Biotechnology). Immunoreactivity was revealed with horseradish peroxidase (HRP) or alkaline phosphatase secondary antibodies (1:5,000, GE Healthcare Bio-Sciences, Piscataway, NJ, USA or Jackson ImmunoResearch Laboratories, West Grove, PA, USA) and ECL or NBT/BCIP (GE Healthcare, EMD Millipore, Billerica, MA, USA, or Promega, Madison, WI, USA).

### RT-PCR analyses of PrP, spliced XBP1, ATF6α and β-actin mRNA

Total RNA was isolated from MCF-7 with the TRIzol reagent according to the manufacturer's protocol (Invitrogen Life Technologies, Burlington, ON, Canada). Reverse transcription was accomplished with avian myeloblastosis virus (AMV) reverse transcriptase (Roche Diagnostics, Laval, QC, Canada) and Oligo dT_12-18 _(GE Healthcare) while the PCR amplification were done with FideliTaq DNA polymerase (USB Corporation, Cleveland, OH, USA). The primers were: PrP-For 5'-GGAACAAGCCGAGTAAGCTAAAA ACCAACATGAAGCAC-3', PrP-Rev 5'-GGTTGTGGTGACCGCGTGCTGCTTGATTG-3', XBP1-For 5'-GGGTCCAAGTTGTCCAGAATGC-3', XBP1-Rev 5'-TTACGAGAGAAAACTCATGGC-3' [47], β-actin-For 5'-CTGGAACGGTGAAGGTGACA-3' and β-actin-Rev 5'-AAGGGACTTCCTGTAACAATGCA-3', ATF6α-For 5' TGGGGGAGTCACACAGCTCCC 3', ATF6α-Rev 5' AGCTGCCGCTTCAGTGTTCCA 3'. The PCR conditions were 1 cycle of 5 minutes at 95°C, 25 cycles of 30 sec at 95°C, 1 minute at 50°C and 1 minute at 68°C, followed by 1 cycle of 5 minutes at 68°C.

### Luciferase assays

The pGL-538 and pGL-214 human PrP promoter constructs were kindly provided by Dr. J. Collinge (MRC Prion Unit, London, UK) and contain 538 or 214 nucleotides upstream of the start site and 125 nucleotides of exon I into the pGL2-Basic vector [48]. The pRL-TK vector was obtained from Promega (Madison). Point mutations in pGL-538 were performed using QuikChange Site-Directed Mutagenesis (Agilent Technologies, Santa Clara, CA, USA). The following forward primers were used to produce point mutations in ERSE-like 5'-AAGATGATTTTTACAGTCAATGAGATCTAG AAGGGAGCGATGGCACCCGCAGG-3', in ERSEa 5'-CGGCCCTGCTTGGCAGCGCGATCGACTTTAACTTAA ACCTCGGC-3', in ERSE-II 5'-GCGCGGCAATTGGTCATATGGCCGACCTCCGCCCGCG-3' and in ERSEb 5'-GCGGCAATTGGTCCCCGCATATGTCTCCGCCCGCGAGCGCCG-3'. The generated mutants were verified by restriction enzyme digestion because the mutations introduced a Pvu*I *site in the ERSEa mutant, Xba*I *and Bgl*II *sites in the ERSE-like mutant and Nde*I *site in the ERSE-II and ERSEb mutants. HEK293T cells (ATCC) seeded at 0.6 × 10^6 ^cells/35 mm well were transfected or co-transfected with 4 μg of pGL-214, pGL-538 or mutated pGL-538 and 0.35 μg of pRL-TK in Lipofectamine™ 2000 reagent (Invitrogen). ER stressors or the DMSO control was added for 6 hrs, 24 hrs after the transfection. Proteins were recuperated as recommended in the Dual-Luciferase Reporter Assay System (Promega). Firefly luciferase and Renilla luciferase activities were measured and expressed as the relative luminescence units (RLU) corresponding to the ratio of the firefly luciferase activity over the Renilla luciferase activity per μg of proteins. Data were normalized to luciferase activity levels obtained from wild type *PRNP *promoter.

### Overexpression or silencing of proteins

Co-transfections of pGL-538 (400 ng/24-well) and 400 ng/24-well pCGN-IRES-EGFP, pCGN-HA-splicedXBP1-IRES-EGFP (both kindly provided by Dr R. Kaufman, U. Michigan), or pCGN-ATF6α (1-373) (Addgene plasmid 27173) [49] with the 75 ng/24-well pRL-TK vector were done with Lipofectamine™ 2000 (Invitrogen) in HEK293 cells for 24 hrs before assessing Firefly and Renilla luciferase with the Dual-Luciferase Reporter Assay System (Invitrogen). Both XBP1 and ATF6α up-regulated the Renilla luciferase promoter so the Firefly luciferase data were expressed relative to the Renilla luciferase of pCGN-EGFP and pRL-TK transfected cells. RT-PCR was conducted to confirm the over-expression of ATF6α and XBP1. For silencing of ATF6α and XBP1, HEK293 cells were co-transfected with 75 ng/24-well of pRL-TK, 800 ng/24-well of pGL-538, and 200 nM siRNAs against human ATF6α (siATF6α) from Santa Cruz Biotechnology (pool of three target specific siRNAs: sc-37699) and from Dharmacon (Dharmacon, Lafayette, CO, USA) (one siRNA: On-TARGETplus human ATF6α (22926) siRNA) or human XBP1 siRNAs from Santa Cruz Biotechnology (pool of three target specific siRNAs: sc-38627) and Dharmacon (one siRNA: On-TARGETplus human XBP1 (7494) siRNA). The si Control (siCtl) was a scrambled siRNA from Santa Cruz Biotechnology (sc-37007) and all transfections were done with Lipofectamine™ 2000. The siRNAs were transfected for 24 hrs and then ER stressors added for 6 hrs before luciferase assays.

For over-expression of proteins in MCF-7 cells, the constructs (4 μg/6-well) were transfected with Lipofectamine™ 2000. The pCep4β-PrP construct was previously described [2], and the pCGN-HA-ATF4-IRES-EGFP construct was a kind gift from Dr R. Kaufman [50]. Proteins were extracted after 24 hrs.

For silencing with siRNAs (at concentrations indicated above), HEK293 cells were transfected with Lipofectamine™ 2000, MDA-MB-231 and HS578T were transfected with Lipofectamine™ RNAiMAX (Invitrogen), and MCF-7 cells were transfected by nucleofection (Amaxa Kit V, VCA-1003, Lonza, Basel, Basel-Stadt, Switzerland) following the manufacturer's protocol, as these methods were the best for each specific cell line. The siRNAs against PrP (siPrP) were human PrP-targeting siRNA from Santa Cruz Biotechnology (sc-36318) or the Dharmacon On-TARGETplus human PrP (5621) siRNA. MCF-7 cells were transfected for 24 hrs before submitting them to an ER stressor for 18 hrs before RNA or protein extraction. MDA-MB-231 and HS578T cells were transfected for 24 hrs before RNA or protein extraction.

### Chromatin immunoprecipitation assay (ChIP)

The ChIP assays were performed as described previously [51] with some modifications. MCF-7 cells treated for three hours with 5 μg/mL BFA or DMSO were harvested by trypsinization and cross-linked in 1% formaldehyde. Nuclei were lysed for 20 minutes on ice in sonication buffer (50 mM Hepes pH 7.4, 140 mM NaCl, 1 mM EDTA, 1% Triton X-100, 0.1% sodium deoxycholate, 1% SDS, protease inhibitors). Nuclear lysates were sonicated and pre-cleared with Protein G-Sepharose (Sigma) pre-coated with 1 μg/mL sonicated salmon sperm nuclei (Sigma), 1 mg/mL BSA. Chromatin was then incubated with pre-coated Protein G-Sepharose and ATF6, XBP1 or control IgG antibodies (3 μg). After washing, the immunoprecipitates were eluted with 50 mM NaHCO_3_, 1% SDS, 1 mM EDTA and 50 mM Tris-HCl pH 8.0 and cross-linking reversed. The DNA was purified and used for PCR amplification.

### Cell death assays

For PrP silencing, siRNAs were transfected for 30 minutes, then ER stress was done for 6 hrs and removed, cell death assays were conducted after another 18 hrs. For the MCF-7 cells, 0.1 μg/mL BFA, 1 μg/mL Thps and 5 μg/mL TM was used. For MDA-MB-231 and HS578T cells, 5 and 10 μg/mL of each ER stressors was used. The chromatin was stained for 20 minutes with 1 μg/mL Hoechst 33342 (Sigma). Caspase activity was measured with the SR Fluorochrome-labeled inhibitors of caspases (FLICA) poly caspase kit (AbD Serotec, Raleigh, NC, USA) according to the manufacturer's instructions.

### Molecular mechanism of PrP protection

Prior to seeding, MCF-7 cells were transfected with scrambled or siPrP by nucleofection (Amaxa Kit V, VCA-1003, Lonza) following the manufacturer's protocol. Cells were treated with ER stressing drugs 5 μg/ml BFA, 1 μg/ml Thps or 5 μg/ml TM for 6 h. Cells were lysed (150 mM NaCl, 50 mM Tris-HCl pH 8.0, 5 mM EDTA pH 8.0, 1% CHAPS or NP-40, 38 μg/mL AEBSF, 0.5 μg/mL Pepstatin A, 1 μg/ml TLCK and 0.5 μg/ml Leupeptin) at 6, 12 or 18 h post-treatment and protein content was quantified using the BCA method. Protein (500 μg) was immunoprecipitated using Protein G-coupled beads and the active Bax-specific 6A7 antibody (1:100 or 5 μg/ml, BD Pharmingen, Mississauga, ON, Canada). Beads were washed, resuspended in loading buffer, boiled and immunoprecipitated Bax levels were assessed by Western blot. Lysates were simultaneously used to investigate BiP, Bax, Bim, Bcl-2 and β-actin levels by Western blot. Proteins from untransfected cells lysed in CHAPS or NP-40-containing lysis buffers acted as negative and positive control for Bax activation.

### Statistical analysis

Previously published data and PrP scores in low and high BiP TMAs were compared with a Student *t*-test two-way assuming equal variance. To compare PrP and BiP scores of each tumor grade, means were compared using non-parametric Kruskal-Wallis test with the InStat 3.1a software (GraphPad Software Inc., La Jolla, CA, USA). Correlations were performed with Spearman's rank correlation with InStat. For all other statistical significance tests an analysis of variance (ANOVA) followed by Scheffé's or Tukey-Kramer *post-hoc *test was performed using StatView software (SAS Institute Inc., Cary, NC, USA). A *p*-value of less than 0.05 was taken as a significant difference.

## Results

### Higher levels of PrP mRNA in breast tumors are associated with a poor prognosis

**Figure 1:**
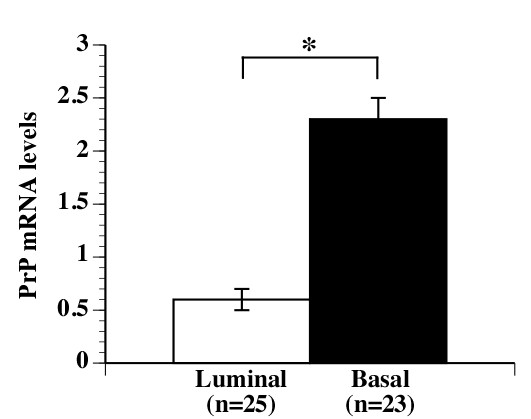
**PrP levels are increased in basal breast carcinoma cell lines**. PrP mRNA levels were averaged in basal or luminal tumor cell lines. Data represent mean ± SEM of PrP. * indicates *p *≤0.05 between both groups.

Data analyses of three publically available breast cancer mRNA microarray databases [52-54] revealed that higher PrP mRNA levels were significantly associated with lower estrogen (ERS) and progesterone (PR) receptor levels and higher tumor grades (Table 1). One study indicated an earlier age of diagnosis in high PrP mRNA tumors [53]. Moreover, a statistically significant association was observed between high PrP levels and metastatic events (Table 1). The percentage of individuals with a metastatic event within five years doubled in the high PrP group and the number of years that individuals remained lung and bone metastasis free decreased significantly by one year in the high PrP group. Analyses of mRNA microarrays of 56 breast carcinoma cell lines [55] showed that basal breast cancer cell lines have higher PrP mRNA levels than luminal cell lines (Figure 1). Together, these results indicate that high PrP mRNA levels are associated with poorer prognosis breast cancers.

**Table 1:** Association between PrP mRNA levels and breast cancer parameters

	Chin *et al*., 2006			van der Vijver *et al*., 2002			Minn *et al*., 2005		
	Low PrP (*n *= 59)	High PrP ( *n *= 59)		Low PrP (*n *= 147)	High PrP (*n *= 147)		Low PrP (*n *= 49)	High PrP (*n *= 49)	
	Mean ± SD	Mean ± SD	*P-*value	Mean ± SD	Mean ± SD	*P-*value	Mean ± SD	Mean ± SD	*P-*value
PrP	0.75 ± 0.1	1.49 ± 0.4	< 0.001*	0.72 ± 0.2	1.53 ± 0.6	< 0.001*	0.63 ± 0.1	1.38 ± 0.4	< 0.001*
ERS	0.81 ± 0.4	0.47 ± 0.5	< 0.001*	0.90 ± 0.3	0.63 ± 0.5	< 0.001*	0.82 ± 0.4	0.33 ± 0.5	< 0.001*
PR	0.68 ± 0.5	0.45 ± 0.5	0.012*	Not Available			0.59 ± 0.5	0.27 ± 0.4	0.001*
Grade	2.29 ± 0.7	2.53 ± 0.7	0.049*	2.06 ± 0.8	2.23 ± 0.8	0.068	Not Available		
Age	55.1 ± 16	55.1 ± 14	0.995	44.5 ± 5	43.5 ± 6	0.129	58.7 ± 14	53.3 ± 12	0.050*

5Y Met Event	% with metastasis after 5 yrs 1 = yes, 0 = no						0.19 ± 0.4	0.44 ± 0.5	0.029*
Met Event	% with metastatic event independent of time						0.24 ± 0.4	0.42 ± 0.5	0.085
MFS	% with metastasis free survival (yrs)						5.63 ± 2.0	4.68 ± 2.5	0.064
LM Event	Lung metastasis						0.05 ± 0.2	0.28 ± 0.5	0.007*
LMFS	Lung metastasis free survival (yrs)						6.02 ± 1.8	4.96 ± 2.6	0.037*
BM Event	Bone metastasis						0.13 ± 0.3	0.21 ± 0.4	0.362
BMFS	Bone metastasis free survival (yrs)						5.83 ± 1.9	4.82 ± 2.5	0.046*

### ER stress is associated with increased BiP and PrP levels in human breast cancer biopsies

**Figure 2:**
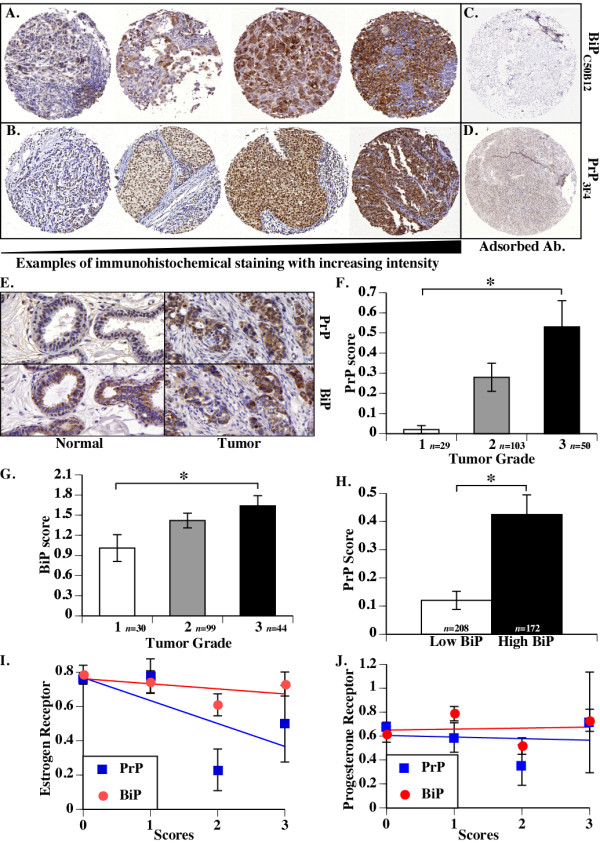
**PrP levels are significantly higher in high BiP-expressing breast cancer tumors**. Representative micrographs of breast tissue cores immunostained for BiP (C50B12) (**A**) or PrP (3F4) (**B**), adsorption control for BiP (**C**) and PrP (**D**). **E **shows staining of PrP and BiP in normal versus tumor tissue cores. Mean intensity score (mean ± SEM) of PrP (**F) **or BiP (**G) **immunostaining correlated with tumor grade. **H**. Comparison of the mean intensity score (mean ± SEM) of PrP immunostaining in breast tissue cores showing Low (0 to 1) or High BiP (2 to 3) immunostaining scores. Correlation between averaged estrogen **(I) **and progesterone (**J**) receptor levels in cores (ERS/PR positive = 1 and ERS/PR negative = 0) correlated with PrP scores. Correlations were done with Spearman's rank correlation in InStat. ERS versus PrP: r = -0.2036, *p = *0.0025, PR vs PrP: r = -0.2277, *P *= 0.0007, ERS vs BiP: r = -0.08819, *P *= 0.2108, and PR vs BiP r = -0.04398, *p *= 0.5353.

To evaluate if ER stress is associated with high PrP levels, we investigated the ER stress marker BiP (also known as GRP78) and PrP levels in breast cancer TMA by immunohistochemistry. PrP was detected in the cytoplasm and in the nucleus of epithelial cells, infrequently found at the cell surface of epithelial cells and was absent in stromal cells (Figure 2A). On the other hand, BiP staining was only found in the cytoplasm, as expected for this ER-resident protein (Figure 2B). Immunoreactivity was eliminated in protein-adsorbed antibodies (Figure 2C, D). The tissue cores were individually analyzed by a trained pathologist (OA) and scored as 0 (absence of staining), 1 (mild staining), 2 (moderate staining) and 3 (high staining). PrP immunostaining was only observed in tumor tissue cores and not in normal tissue cores whereas BiP was present in both normal and tumor tissue cores (Figure 2E). Higher PrP and BiP levels segregated with a higher tumor grade (Figure 2F, G). The high BiP-expressing tumor cores had higher PrP levels than low BiP-expressing tumors indicating that ER stress is associated with increased PrP in these tissues (Figure 2H). Furthermore, the levels of PrP correlated significantly with the loss of estrogen receptor (Spearman r = -0.2036, *P *= 0.0025) and progesterone receptor (Spearman r = -0.2297, *P *= 0.0007) negative tumors (Figure 2I, J). In contrast, no significant correlation was observed between the levels of BiP and the estrogen and progesterone receptor status. Supporting these results, analysis of the mRNA microarray data [52-54] indicated that in two of three studies, high-BiP expressing tumors were associated with higher PrP mRNA levels (Table 2). Furthermore, higher BiP mRNA in breast carcinoma cell lines correlated with a higher PrP mRNA level (Table 2). Overall, these results suggest that ER stress is associated with increased *PRNP *gene expression in human breast cancer tumors with a basal subtype and poor outcome in human patients.

**Table 2:** Association between high BiP mRNA levels and PrP mRNA levels

		Low BiP	High BiP	*P-*	*n*	Reference
		Mean ± SD	Mean ± SD	value		
	BiP	0.80 ± 0.1	1.29 ± 0.3	≤0.001*	127	van der Vijver *et al*., 2002
	PrP	1.01 ± 0.5	1.24 ± 0.7	0.002*		
	
Breast cancer	BiP	0.78 ± 0.1	1.26 ± 0.3	≤0.001*	44	Minn *et al*., 2005
	PrP	0.93 ± 0.4	1.09 ± 0.5	0.1		
	
	BiP	0.76 ± 0.2	1.43 ± 0.4	≤0.001*	40	Chin *et al*., 2006
	PrP	1.00 ± 0.5	1.38 ± 0.6	0.003*		

Breast carcinoma	BiP	0.77 ± 0.2	1.37 ± 0.4	≤0.001*	56	Neve *et al*., 2006
cell lines	PrP	0.99 ± 0.9	1.94 ± 1.0	0.001*		

### ER stress transcriptionally increases PrP levels in MCF-7 cells

**Figure 3:**
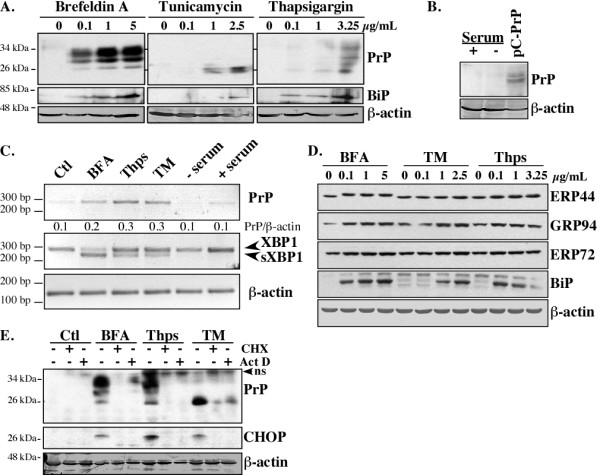
**ER stress transcriptionally increases PrP levels in MCF-7 cells**. One representative Western blot from at least three independent experiments is shown in **A-B**. (A) Western blots of PrP with the 3F4 antibody, BiP, and β-actin in protein extracts from MCF-7 cells treated 18 hrs with increasing concentrations of Brefeldin A, Tunicamycin, or Thapsigargin. (B) Western blot of PrP (3F4) and β-actin in protein extracts from MCF-7 cells incubated for 18 hrs in the absence or presence of serum or transfected with pCep4β-PrP (pC-PrP). **(C) **PrP, XBP1, sXBP1 and β-actin RT-PCR cDNA amplicons from cells treated for 6 hrs. The ratio of PrP over β-actin was calculated from three independent experiments. (**D**) Western blot of various ER stress-regulated proteins in MCF-7 cells treated 18 hrs with BFA, TM or Thps. **(E) **Western blot of PrP with the 3F4 PrP, CHOP and β-actin antibodies in protein extracts from MCF-7 cells treated 18 hrs with DMSO (Ctl) or ER stressors in the presence or in absence of cycloheximide (CHX) or actinomycin D (Act D). The immunoreactive band at 37 kDa was not consistently detected with the anti-PrP 3F4 antibody suggesting a non-specific band (ns).

To experimentally assess if ER stressors up-regulate *PRNP *gene expression in breast cancer cells, we assessed the luminal subtype breast carcinoma MCF-7 cell line, which lacks endogenous PrP expression under steady state conditions. MCF-7 cells were exposed to three different ER stressors: Golgi-disaggregating BFA, N-linked glycosylation inhibitor Tunicamycin (TM), and ER Ca-ATPase family (SERCA) inhibitor Thapsigargin (Thps). Each ER stressor increased PrP levels in a dose-dependent manner within 18 hrs of treatment (Figure 3A). PrP accumulated as immature glycosylated proteins (28 to 33 kDa) with BFA, as unglycosylated (25 kDa) protein with TM, and as unglycosylated, immature, and mature (34 to 36 kDa) glycosylated proteins with Thps. Increased BiP protein levels confirmed induction of the ER stress response with the BFA, TM and Thps treatments. The ER stress-mediated up-regulation of PrP appeared specific since serum-deprivation of MCF-7 cells for 18 hrs did not increase PrP levels (Figure 3B). ER stressors increased PrP mRNA levels within six hours of treatment in MCF-7 cells with concomitant splicing of XBP1 (sXBP1), although sXBP1 was more prominent in the BFA-treated MCF-7 cells (Figure 3C). ER stress was further confirmed by Western blots showing an increase of ERP44, GRP94, ERP72 and BiP, proteins known to be up-regulated in ER stress (Figure 3D). Furthermore, the transcriptional inhibitor actinomycin D (Act D) or the translational inhibitor, cycloheximide (CHX) strongly inhibited the ER stress-mediated increase of PrP levels and the increase of the ER stress-related protein CHOP (Figure 3E). Together, these results show that ER stress of the luminal breast carcinoma MCF-7 cell line increases *PRNP *gene transcription.

### *PRNP *gene expression is up-regulated by ER stress in basal carcinoma cell lines

**Figure 4:**
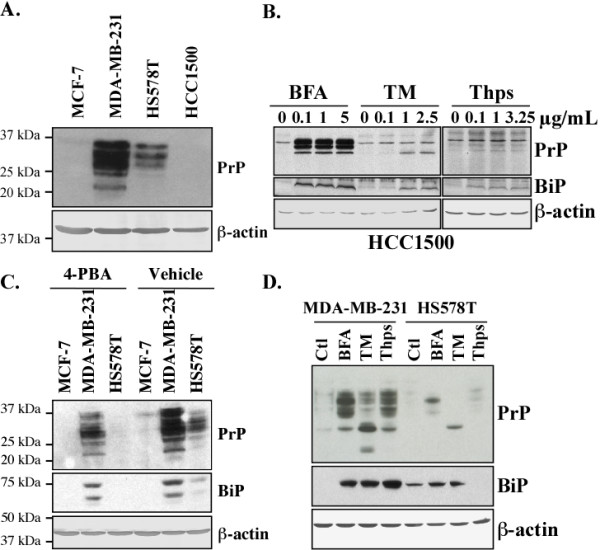
**ER stress increases PrP levels in MDA-MB-231, HS578T and HCC1500 cell lines**. Western blot of (**A) **PrP in protein extracts of MCF-7, MDA-MB-231, HS578T and HCC1500 cell lines, (**B) **PrP and BiP in protein extracts from HCC1500 cells treated with BFA, TM or Thps, (**C**) PrP and BiP in protein extracts from MCF-7, MDA-MB-231 and HS578T cell lines treated with 4-PBA or vehicle, (**D**) PrP and BiP in protein extracts of MDA-MB-231 and HS578T cell lines treated with BFA, TM and Thps. β-actin was probed as a loading control.

A comparison of basal breast carcinoma cell lines, MDA-MB-231, HS578T and HCC1500, with luminal MCF-7 cells revealed a high level of PrP in the MDA-MB-231 and HS578T cell lines but not in the HCC1500 basal cell line. However, BFA, TM and Thps increased the levels of PrP and BiP in the HCC1500 cell line (Figure 4B) as observed in the luminal MCF-7 cell line (Figure 3A). Treatment of MDA-MB-231 and HS578T cells with 4-phenyl butyric acid (4-PBA), an inhibitor of ER stress [56], decreases both BiP and PrP levels suggesting that PrP is increased by intrinsic ER stress in these two basal carcinoma cell lines (Figure 4C). Nevertheless, the three pharmacological ER stressors further increased both BiP and PrP levels in MDA-MB-231 and HS578T cells (Figure 4D). Together, these results show that intrinsic or exogenous ER stress up-regulates *PRNP *gene expression in basal breast carcinoma cell lines.

### Identification of four ER stress response elements in PRNP promoter

**Figure 5:**
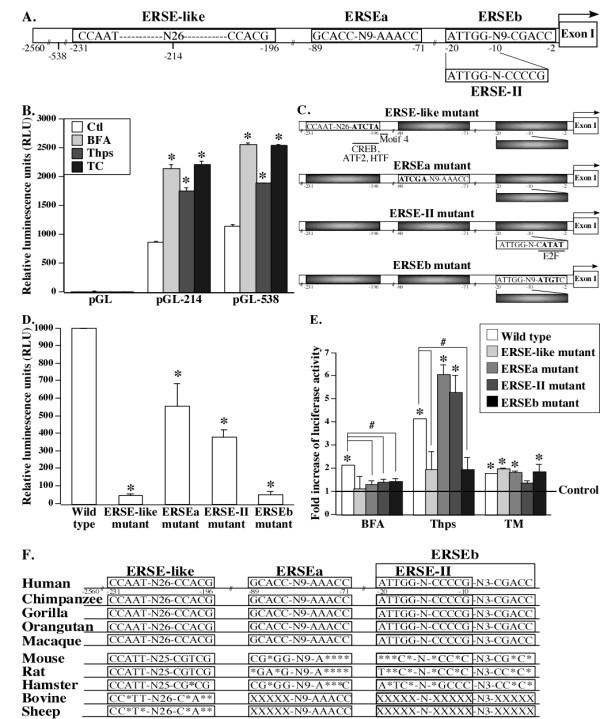
**Identification of ER stress response elements (ERSE) in the *PRNP *promoter**. (**A**) Schematic diagram of two ERSE (ERSEa and ERSEb), one ERSE-like, and one ERSE-II in the human *PRNP *promoter. (**B**) Luciferase activity in HEK293T cells transfected with pGL2 (empty vector), pGL-214 (vector containing the first 214 nucleotides of human *PRNP *promoter), or pGL-538 (first 538 nucleotides of human *PRNP *promoter) and treated six hours with ER stressors. Data are expressed as the mean ± SD of two experiments done in triplicate. * Indicates *P *≤0.05 compared to the control (Ctl). (**C**) Schematic diagram of the *PRNP *promoter mutants showing the mutated nucleotides in bold. Putative transcription factor binding sites predicted by TRANSFAC and the conserved motif 4 affected by the mutations are shown. (**D) **Luciferase activity measured in HEK293T cells transfected with wild type *PRNP *promoter (pGL-538) or ERSE mutants of the *PRNP *promoter. Data represent the mean ± SEM of three experiments done in triplicate. * Indicates *P *≤0.05 compared to wild type. (**E**) Luciferase activity in HEK293T cells transfected with wild type or ERSE mutant *PRNP *promoters and treated with DMSO (control) or ER stressors. The fold increase of luciferase activity calculated for each *PRNP *promoter construct corresponds to the ratio of the RLU in presence of ER stress over the RLU in presence of DMSO (Control). Data represent the mean ± SEM of three independent experiments done in triplicate. * Indicates *P *≤0.05 compared to the control (DMSO) and # indicates *P *≤0.05 between the mutants and the wild type *PRNP *promoter. (**F**) Schematic diagram showing conservation of human ERSE-like, ERSEa, ERSE-II and ERSEb. Nucleotide sequence alignment of *PRNP *promoters is from a previous study [48] and from an alignment done with Ensembl databases. N indicates the number of nucleotide. Compared to human *PRNP *promoter, * indicates a non-conserved and non-complementary nucleotide while X denotes an absent nucleotide.

We identified three potential ER stress response elements (ERSE) and one ERSE-II motif in the human *PRNP *promoter using the transcription factor database TRANSFAC and by manually scanning the *PRNP *promoter (EMBL accession no. AJ289875) [48] (Figure 5A). No consensus UPR element (UPRE) or amino-acid-regulatory element (AARE) was found in the *PRNP *promoter. Two ERSE motifs, named here ERSEa and ERSEb to easily discriminate between them, were located at nucleotides -89 to -71 and at -20 to -2, respectively. ERSEa was in the opposite orientation and had a T to A substitution (CCAAT to CCAAa) compared to the ERSE consensus sequence. The ERSEb nucleotide sequence also contained two CG substitutions (CCACG to CgACc). One non-conventional ERSE (ERSE-like) motif, located from -231 to -196 and predicted by TRANSFAC, contained N26 rather than the canonical N9 of the glucose-regulated proteins CCAAT-N9-CCACG ERSE consensus sequence [35]. One canonical ERSE-II motif was contained within ERSEb (-20 to -10). Compared to the ERSE-II consensus sequence (ATTGG-N-CCACG), the ERSE-II motif in the *PRNP *gene promoter had an A to C substitution (CCACG to CCcCG).

*PRNP *transcriptional activation was assessed with the *PRNP *promoter-luciferase reporter constructs pGL-214 and pGL-538, which contain 214 or 538 nucleotides preceding the major transcriptional start site of *PRNP*, as well as 125 nucleotides of the non-coding exon I [48]. Under normal conditions, substantial luciferase activity was detected in pGL-214- and pGL-538-transfected HEK293T cells, compared to the pGL2 empty vector-transfected cells (Figure 5B). The three ER stressors increased the luciferase activity in pGL-214- and pGL-538-transfected cells by 1.7- to 2.6-fold. Point mutations in the ERSE-like, ERSEa, ERSE-II and ERSEb motifs (Figure 5C) considerably reduced the constitutive *PRNP *promoter activity (Figure 5D). This has also been observed with other ER stress regulated gene promoters [35,42,43,57] and may be explained by the presence of transcriptional motifs overlapping the ERSE as indicated in Figure 4C. Alternatively, the mutations affect the structure of the promoter rendering it less responsive to transcriptional factors. Cells treated with BFA lost their ability to significantly induce luciferase activity in the *PRNP *ERSE mutants (Figure 5E). The ERSE-like and ERSEb mutations abolished the induction of luciferase activity in Thps-treated cells, while it was retained in the ERSEa and ERSE-II mutants. TM induced luciferase activity in the cells transfected with wild type or mutant ERSE-like, ERSEa and ERSEb *PRNP *promoter but not in the ERSE-II mutant. The four ERSE motifs of the human *PRNP *promoter are well conserved among primates (Figure 5F), but not well conserved in other mammalian species. Only the ERSE-like is highly conserved in the mouse, rat or hamster *Prnp *promoters.

Taken together, these results indicate that (a) the *PRNP *promoter ERSE motif sequences overlap with those regulating the basal expression of *PRNP*, (b) the ERSE motifs positively regulate ER stress-mediated transcription of the *PRNP *gene, (c) the *PRNP *promoter is differentially regulated with the three different ER stresses, and (d) ER stress-mediated regulation of *PRNP *gene expression may have evolved in primates but not in other mammalian species.

### Role of ATF6α and XBP1 in ER stress-mediated PRNP gene expression

**Figure 6:**
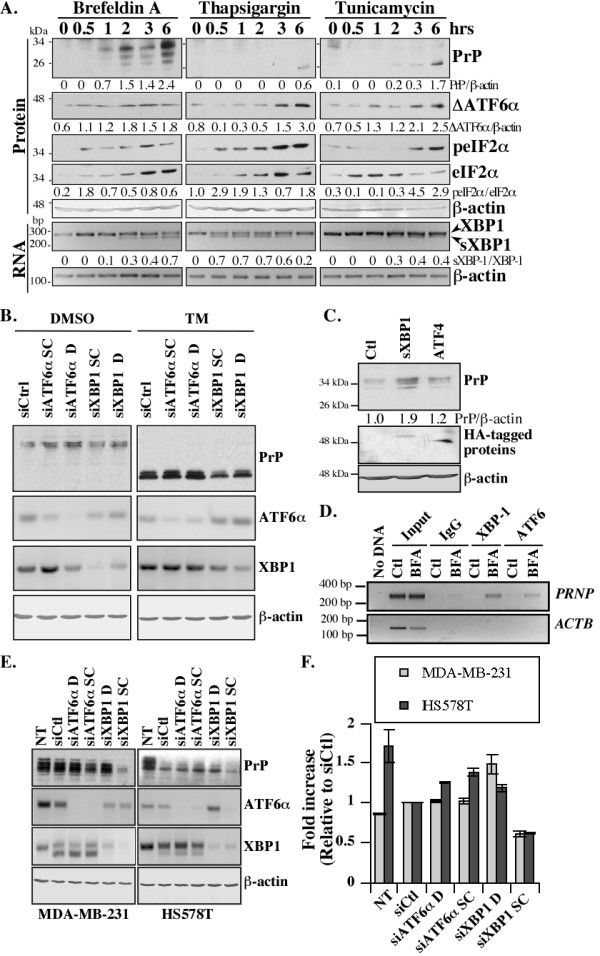
**sXBP1 is involved in *PRNP *gene expression in MCF-7 and MDA-MB-231 cells**. **(A) **Western blot analyses of PrP with the 3F4 antibody, cleaved ATF6α (ΔATF6α), phosphorylated eIF2α (peIF2α), total eIF2α, and β-actin. Lower panels represent XBP1, spliced XBP1 (sXBP1) and β-actin amplified by RT-PCR. Protein or mRNA extracts were from MCF-7 cells treated with ER stressors for 0 to 6 hrs. The increase of PrP and ΔATF6α levels compared to β-actin levels and the ratios of peIF2α/eIF2α and sXBP1/XBP1 are indicated. **(B) **MCF-7 cells transfected with siATF6α or siXBP1 and proteins immunoblotted with 3F4 PrP and β-actin antibodies. RT-PCR shows levels of ATF6α and XBP1 mRNAs. **(C) **Western blot analyses of PrP, HA tag and β-actin in protein extracts from MCF-7 cells transfected for 6 hrs with pCGN-IRES-EGFP (Ctl), pCGN-HA-sXBP1-IRES-EGFP, and pCGN-HA-ATF4-IRES-EGFP constructs. **(D) **ChIP assays performed on DMSO (Ctl)- or BFA-treated MCF-7 cells with IgG control, XBP1 or ATF6α antibodies. PCR amplification of *PRNP *and β-actin gene promoters (*ACTB*) was done on immunoprecipitated and non-immunoprecipitated (input) DNA. (**E**) Western blot of PrP (top panel) and β-actin (bottom panel) and ethidium stained agarose gel containing ATF6α and XBP1 amplicons from MDA-MB-231 or HS578T cells transfected with siCtl, siATF6α or siXBP1. NT indicates non-transfected, D indicates the Dharmacon siRNAs and SC indicates the Santa Cruz siRNAs. (**F) **Levels of *PRNP *mRNA detected by qRT-PCR in siATF6α or siXBP1-transfected cells.

To determine which transcription factor regulates ER stress-mediated *PRNP *gene expression in MCF-7 cells, we investigated ΔATF6α, sXBP1 and phosphorylation of eIF2α (peIF2α), as markers of the three UPR response pathways, and compared these to PrP levels. Because the ATF4 motif is absent in the *PRNP *promoter, we focused on ΔATF6α and sXBP1. In MCF-7 cells, ΔATF6α and peIF2α appeared within 30 minutes, sXBP1 increased between one to two hours, and PrP levels increased within one hour of BFA treatment (Figure 6A). With Thps, truncated ATF6α increased at three hours, peIF2α and sXBP1 increased within 30 minutes, and PrP levels increased at six hours of treatment. TM induced truncated ATF6α within one hour, peIF2α within three hours, and sXBP1 and PrP within two to three hours. Variation in total eIF2α levels was observed under the three ER stressors, as previously reported [58,59], while β-actin mRNA and protein levels remained relatively constant. These results indicate that all three UPR response pathways are activated before ER stress-mediated PrP up-regulation in MCF-7 cells.

Silencing ATF6α with two different siRNAs did not alter TM-mediated increased levels of PrP (Figure 6B). However, knock down (KD) of XBP1 in MCF-7 cells, considerably stunted the TM-mediated increase in PrP. Over-expression of HA-tagged sXBP1 increased PrP levels above low levels observed in cells transfected with the control pCGN-IRES-EGFP vector, while HA-tagged ATF4 did not modulate PrP levels (Figure 6C). Using chromatin immunoprecipitation (ChIP) assays, we further showed that both XBP1 and ATF6α bound to the *PRNP *promoter in BFA-treated MCF-7 cells (Figure 6D). In contrast, neither interacted with the β-actin *ACTB *promoter. These results show that sXBP1 in MCF-7 cells is involved in transactivating *PRNP *gene expression, whereas ATF6α can interact with, but does not transactivate *PRNP*.

To determine if *PRNP *gene expression in the basal carcinoma cell lines depends on ER stress, we knocked down (KD) ATF6α and XBP1 with siRNAs in the MDA-MB-231 and HS578T cell lines (Figure 6E). In the MDA-MB-231 cell line, two different siRNAs to ATF6α and XBP1 efficiently decreased the levels of ATF6α and sXBP1, respectively (Figure 6E). The level of PrP (Figure 6E) and *PRNP *mRNA (Figure 6F) were not decreased by the ATF6α KD. However, the siCtl and siATF6α produced an unexpected increase in sXBP1 in MDA-MB-231 cells, which could obscure the effect of siATF6α on *PRNP *gene expression. Indeed, an almost complete XBP1 KD with siXBP1 SC decreased PrP and *PRNP *mRNA levels by 40%, whereas a partial siXBP1 D KD did not decrease *PRNP *gene expression at either the protein or mRNA level (Figure 6E, F). Both pairs of siRNAs against ATF6α and XBP1 were highly efficient in the HS578T cell line and the siCtl or siATF6α had much less effect on the levels of sXBP1 (Figure 6E). The levels of PrP assessed by Western blotting decreased relative to the non-transfected cells but not necessarily relative to the siCtl, except for the siATF6α SC (Figure 6E). Furthermore, qRT-PCR results showed that the levels of PrP mRNA decreased by 40% only with the siXBP1 SC (Figure 6F). The discrepancy between the effect of these two different siXBP1s is unclear. Both decrease XBP1 mRNA levels similarly so we must assume that one of these has off-target effects that differentially regulate *PRNP *gene expression. These results indicate that the ATF6α is not regulating *PRNP *gene expression in MDA-MB-231 or HS578T cell lines, while XBP1 is possibly implicated in the MDA-MB-231 cell line.

### sXBP1 and ΔATF6α regulate luciferase activity from the pGL-538 PRNP promoter in HEK293 cells

**Figure 7:**
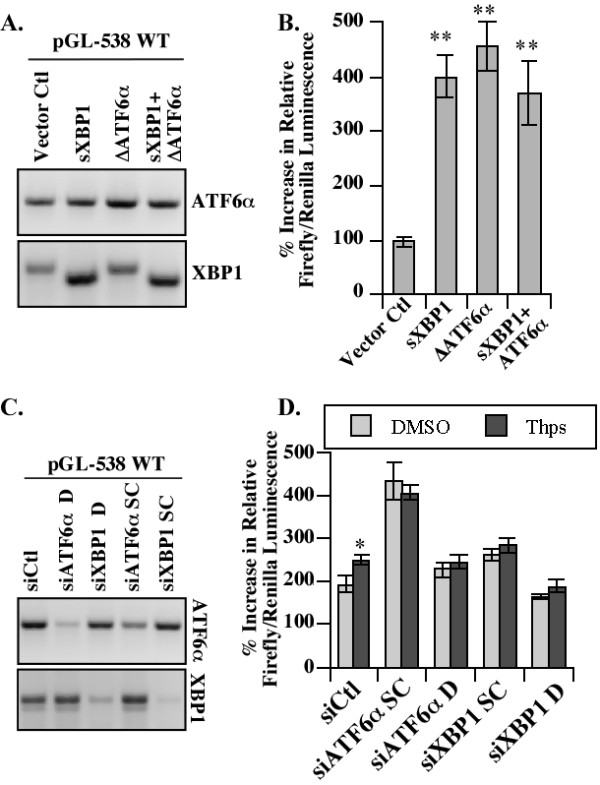
**ATF6α and XBP1 transactivate *PRNP *promoter**. (**A**) Ethidium bromide stained gel showing ΔATF6α, sXBP1 and XBP1 amplicons from HEK293 cells co-transfected with the pGL-538 *PRNP *promoter luciferase reporter and pCGN-ATF6α (1-373) and/or pCGN-sXBP1. (**B**) Luciferase luminescence generated from HEK293 cells co-transfected as described in A. (**C) **Ethidium bromide stained gel showing ATF6α and XBP1 amplicons from HEK293 cells co-transfected with the pGL-538 *PRNP *promoter luciferase reporter and siRNAs against ATF6α or XBP1. **(D) **Luciferase luminescence generated from HEK293 cells co-transfected as described in C. Statistical analyses on B and D were one-way ANOVA followed by a Tukey-Kramer multiple comparison test.

To more directly assess if sXBP1 and ΔATF6α transactivate *PRNP *gene expression from its promoter, HEK293 cells were co-transfected with pCGN constructs encoding sXBP1 or ΔATF6α and the pGL-538 *PRNP *promoter luciferase reporter construct. Overexpression of both ΔATF6α and sXBP1 was confirmed by RT-PCR (Figure 7A) and both of these transcription factors transactivated luciferase expression from the pGL-538 *PRNP *promoter luciferase reporter construct (Figure 7B). Furthermore, KD of ATF6α and XBP1 expression with two different siRNAs (Figure 7C), prevented Thps-induced luciferase expression from the pGL-538 construct (Figure 7D). The siATF6α SC siRNA induced an overall increase in luciferase activity compared to the siCtl but the levels did not differ between DMSO and Thps. This suggests that this siRNA may induce other transcription factors that up-regulate *PRNP *gene expression. Taken together, these results confirm that both ATF6α and sXBP1 can up-regulate *PRNP *gene expression in HEK293 cells.

### PrP delays ER stress-mediated cell death

**Figure 8:**
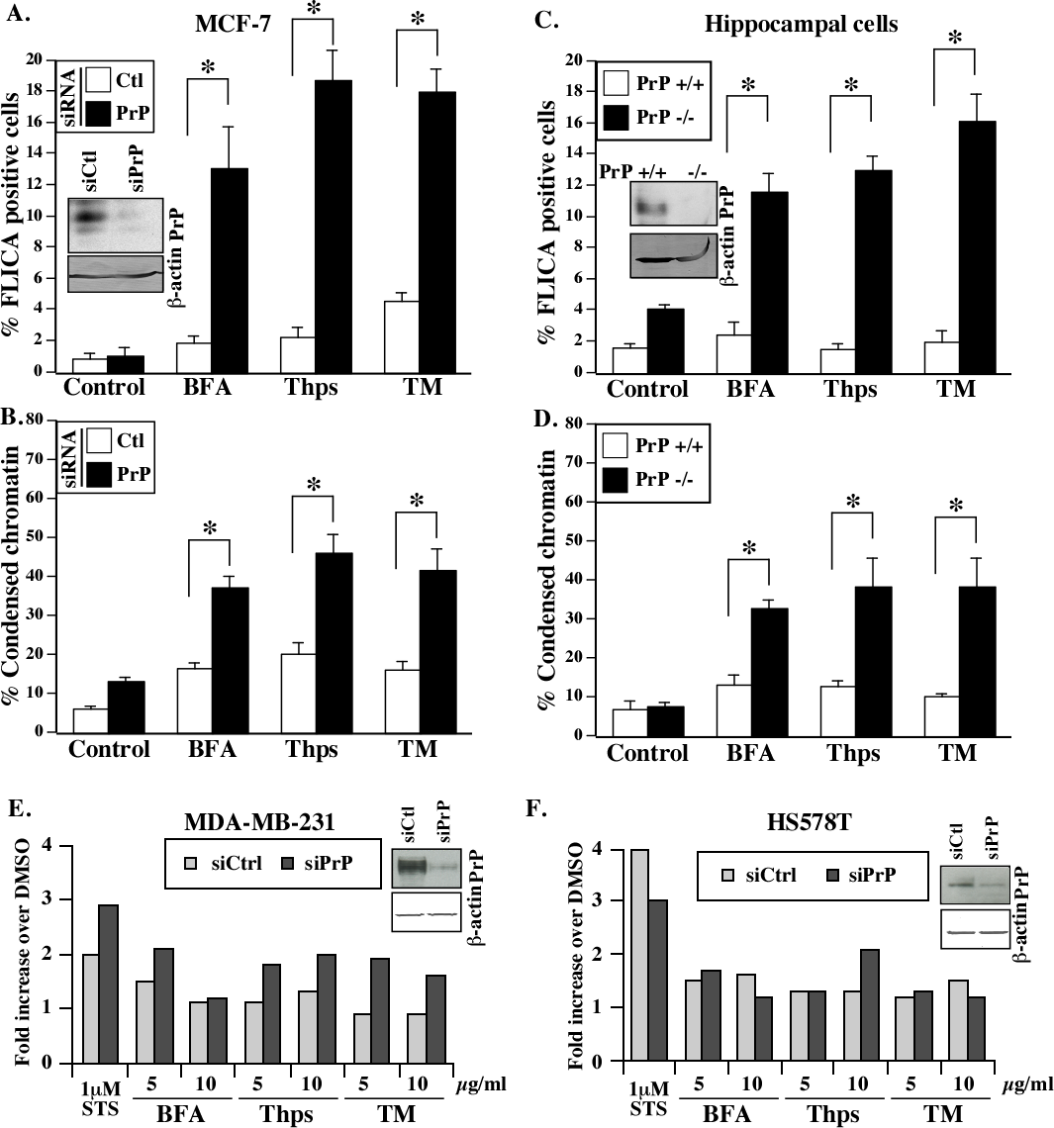
**PrP delays cell death induced by ER stress**. **(A) **inset shows western blot of PrP and β-actin in siRNA transfected cells. Percentage of cell death assessed by pan-caspase FLICA (A) or by chromatin condensation **(B) **in MCF-7 cells transfected with control (siCtl) or PrP (siPrP) siRNAs for 24 hrs and treated with ER stressors or DMSO (control) during 6 hrs. Cell death was measured 18 hrs after the removal of ER stress. Data represent the mean ± SEM of three (A) or six (B) independent experiments. At least 150 cells were counted per experiment. * Indicates *P *≤0.05 between PrP siRNA and control siRNA. **(C-D) **Percentage of cell death assessed by FLICA staining for active caspases (C) or by chromatin condensation in PrP^+/+ ^and PrP-/- hippocampal cell lines (D). Data represent the mean ± SEM of four independent experiments. At least 150 cells were counted per experiment. * Indicates *P *≤0.05 between PrP+/+ and PrP-/- cells. C Inset: Western blot analysis of PrP with the R155 antibody and β-actin in protein extracts from mouse PrP+/+ and PrP-/- hippocampal cell lines. PrP-/- hippocampal cell lines were treated with 2.5 μg/ml of ER stressors. These concentrations were empirically determined to induce ER stress response without initially inducing strong toxic effects. **(E) **Relative fluorescence units (RFU) of FLICA activity representing pan-caspase activity in MDA-MB-231 (E) or HS578T **(F) **cells transfected with siCtl or siPrP.

One way PrP could favor cancer progression is by opposing cell death. Because the cellular form of PrP has anti-apoptotic activity, we investigated if PrP up-regulation could participate in the UPR pro-survival response. Compared to control siRNA, PrP silencing increased the percentage of cells displaying pan-caspase activity (Figure 8A) and condensed chromatin (Figure 8B) in MCF-7 cells treated with BFA, Thps or TM. A similar vulnerability against ER stressors was observed in hippocampal *Prnp *null cells (Figure 8C, E). Furthermore, siRNA KD of PrP increased the levels of pan-caspase activity in MDA-MB-231 cells treated with the three ER stressors, indicating a protection by PrP in this basal cell line (Figure 8E). However, we did not observe higher caspase activity in the HS578T cell line, except in the 10 μg/ml Thps condition (Figure 8F). This result indicates that different cell lines require different doses of ER stress for induction of cell death. Nevertheless, the results indicate that PrP delays ER stress-induced cell death in MCF-7 and in MDA-MB-231 cells.

### PrP does not prevent ER stress-mediated Bax activation in MCF-7 cells

**Figure 9:**
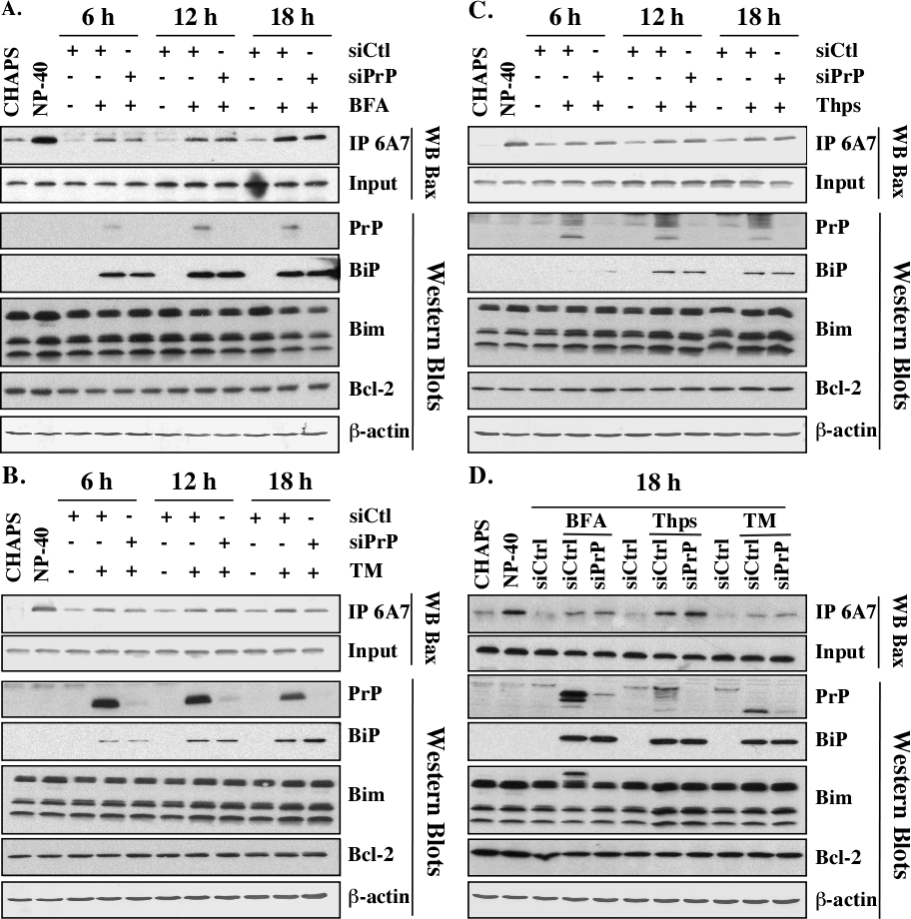
**PrP does not prevent ER stress-mediated Bax activation in MCF-7 cells**. Western blot assessing the impact of PrP silencing on Bax activation as well as Bim and Bcl-2 levels at 6, 12 or 18 h following a 6-h treatment with Brefeldin A **(A)**, Tunicamycin **(B)**, or Thapsigargin **(C)**. Western blot using the 3F4 antibody confirms ER stress-induced increase in PrP levels as well as its efficient siRNA-mediated silencing, while probing for Bax and β-actin controls for equal protein input and protein loading, respectively. (**D) **MCF-7 cells transfected with a second siPrP and treated for 18 h with ER stressor; experiments performed as described in A-C.

We have previously discovered that PrP can inhibit Bax-mediated cell death [2,4]. Immunoprecipitation of the pro-apoptotic 6A7-immunoreactive form of Bax indicated that BFA, TM and Thps all induce Bax activation (Figure 9A-C). However, silencing PrP before ER stress did not increase the level of Bax activation indicating that PrP cannot prevent ER stress-induced Bax activation and that PrP delays ER stress-mediated cell death by another mechanism.

ER stress with BFA, TM or Thps was confirmed by up-regulation of BiP levels in the presence or absence of PrP KD (Figure 9A-C). ER stress is known to induce CHOP during apoptosis and CHOP transcriptionally increases pro-apoptotic protein, Bim [60]. Bim was increased in Thps-treated MCF-7 cells (Figure 9C), but not in BFA or TM-treated cells (Figure 9A, B). Again, siRNA inhibition of ER stress-mediated PrP gene expression did not alter Bim levels. Bcl-2 levels were also constant in ER stress-treated cells with or without PrP silencing with siRNAs. We repeated these experiments with another siRNA against PrP and observed identical results (Figure 9D). Therefore, it is unlikely that PrP protects against ER-mediated cell death by targeting members of the Bcl-2 family of proteins.

## Discussion

In the present manuscript, we show that (1) human *PRNP *gene expression is up-regulated by ER stress, (2) that the UPR-activated sXBP1 and ΔATF6α are involved in transcriptional activation of *PRNP*, (3) that PrP protects against ER stress-mediated cell death, and (4) that PrP may contribute to increased survival of breast cancers.

Our first observation in this study was that *PRNP *gene expression is regulated by ER stress in both basal and luminal breast cancer cell lines. Indeed, three different ER stressors increase the levels of PrP in MCF-7 cells and in the MDA-MB-231, HS578T and HCC1500 breast carcinoma cell lines. The MDA-MB-231 and HS578T cell lines have intrinsic ER stress activity based on higher levels of ER stress-regulated BiP and correspondingly have much higher PrP levels than the MCF-7 and HCC1500 cell lines. Furthermore, the ER stress inhibitor, 4-PBA, decreases both BiP and PrP in MDA-MB-231 and HS578T cell lines. While the overexpression of PrP definitely has a negative impact on several types of cancers, surprisingly little is known about the regulation of *PRNP *gene expression. Human *PRNP *is localized on chromosome 20 and contains a short un-translated exon I separated from the coding exon II by a 13 kb intron [48,61-63]. *PRNP *has one major transcriptional start site and its promoter contains a CCAAT box, a high G/C content, and an SP1 transcription factor binding site characteristic of constitutively expressed genes. It also contains several putative conditionally activated transcriptional binding sites: developmentally-regulated GATA, AP-2, Nkx2-5 and Myo D, signal-dependent p53 and HSE, cell membrane receptor-dependent AP1, immune-mediated nuclear factor IL-6, NF-AT and Ets-1, and metal responsive element binding sites [48,62,64]. These transcriptional binding site sequences are located in two clusters, one within exon I, and the other in the 800 nucleotides upstream of the start site. Of these, only Sp1, metal transcription factor-1 (MTF-1), HSE and p53 recently have been confirmed to regulate human *PRNP *or mouse *Prnp *gene expression [21,64-66]. In addition, four motifs with unknown function are highly conserved among mammalian *PRNP *promoters [48,67]. Our finding that ER stress can up-regulate *PRNP *gene expression has certain implications in cancer because cancers are ER stressed [68] and PrP has been reported to have protective functions that appear to be involved in the resistance of cancers to chemotherapy [12-14,69].

Our second observation is that the up-regulation of PrP occurs through transactivation of the *PRNP *gene. We describe four ERSE in the promoter of *PRNP*, which respond to the three pharmacological ER stressors, BFA, Thps and TM. Mutagenesis of these ERSE dampens the ER stress-mediated up-regulation of *PRNP*. In addition, ΔATF6α and sXBP1 directly increase expression from the *PRNP *promoter and siRNAs against either ATF6α or XBP1 prevents Thps-mediated *PRNP *gene expression. Furthermore, ChIP analyses shows that both XBP1 and ATF6α interact with the *PRNP *promoter. In MCF-7 cells, the three pharmacological ER stressors increase ATF6α and sXBP1 but only the KD of XBP1 prevents ER stress-mediated up-regulation of *PRNP *gene expression. A similar effect is observed in MDA-MB-231 cells but not in the HS578T cell line. These results suggest that *PRNP *gene expression can be regulated by both ATF6α and XBP1, but that the regulation is complex and depends on the cellular context. Together, these results add ER stress as an important human regulator of *PRNP *gene expression.

Interestingly, the ERSE elements are specific to primate *PRNP *promoters since three of the ERSE elements are quite degenerate in rodents and ovines while the remaining ERSE is conserved in rodents but not in ovines. In addition, *PRNP *gene expression is differentially regulated by each of the three ER stressors. The presence of these ERSE in the primate *PRNP *promoter and the differential regulation by ER stressors suggests that the regulation of *PRNP *gene expression is highly complex in primates and is consistent with the reported inability of ER stress to increase PrP levels in murine cells [70-72]. It must, therefore, be noted that ER stress regulation of *Prnp *gene expression would remain undetected in mouse models of disease.

Our third finding is that increased ER stress-mediated *prnp *gene expression protects cells from ER stress-mediated cell death. This is shown by KD of PrP in MCF-7 and MDA-MB-231 cells and in PrP null hippocampal cell lines. However, PrP does not protect HS578T cells from ER stress mediated cell death. These results indicate that the protective function of PrP may not be universal to breast carcinoma cells and that other pathways are involved. This is not unexpected given the complexity of cancers. Previous studies have shown that KD of PrP restores the susceptibility of TRAIL resistant MCF-7 cell lines [12] and over-expression of PrP induces MCF-7 resistance to TNF-α-mediated cell death [13]. These results are entirely consistent with the previously reported protective role of PrP in MCF-7 cells [4,12,13].

However, the mechanism by which PrP protects against ER stress is unclear here. We had previously shown that PrP could prevent Bax-mediated cell death in MCF-7 cells and in human primary neurons [2,3], by preventing the conversion of Bax to pro-apoptotic Bax [4]. However, while we observe the activation of Bax with ER stress in these experiments, silencing PrP does not increase levels of Bax activation. Others have reported ER stress protective mechanisms against estrogen-deprived breast cancer cell death. One of the proposed mechanisms involves BiP directly. Zhou and colleagues identified that BiP protects MCF-7/BUS-10 estrogen starvation resistant cells by interacting with the BH3 domain pro-apoptotic protein Bik, thus allowing the release of Bcl-2 from Bik and increased survival via inhibition of Bax [73]. Other studies show that BiP can regulate cell death via both caspase-dependent and independent cell death [74], and that IGFBP-3 acts as a survival factor in the HS578T cell line via BiP and autophagy [75,76]. However, we show that PrP KD, while increasing protection against ER stress in MCF-7 cells, does not alter the levels of BiP. Therefore, we can exclude BiP as a direct regulator of survival through PrP. Together these results suggest that multiple protective pathways are involved in increasing breast cancer cell resistance to cell death.

Lastly, we describe that the ER stress-mediated increase in PrP levels is associated with increased cellular survival in human breast cancers. Our TMA analyses of human breast carcinomas showed that (a) high ER stress marker BiP levels were associated with higher PrP levels, (b) both BiP and PrP levels were higher in grade 3 tumors compared to grade 1 tumors, and (c) levels of PrP correlate with estrogen and progesterone receptor negative tumors. In three independent mRNA microarray analyses [52-54], higher levels of PrP mRNA were significantly associated with estrogen receptor negative cancers consistent with the previously reported higher resistance to adjuvant chemotherapy in estrogen receptor negative and PrP positive cancers [14]. Additionally, (a) two of the microarray analyses showed a significant reduction of progesterone receptor levels in high PrP expressing cancers [53,54], (b) one study showed a significant association of high PrP mRNA levels with tumor grade [54], and (c) one study showed a lower age at diagnostic [53] and an increase in metastatic events in high PrP mRNA tumors [53]. Furthermore, microarrays show that increased PrP mRNA levels are associated with ER stress in the most aggressive basal breast carcinoma cell lines compared with the less aggressive luminal cell lines ([55] and Figure 1). These results are consistent with findings that estrogen receptor positive breast cancers that are resistant to tamoxifen were shown to have increased sXBP1, indicating that increased ER stress conditions promote survival of luminal cancers [68]. These results indicate that higher PrP levels are associated with a poor outcome in breast cancer.

## Conclusions

Together, these results indicate that ER stress increases levels of PrP that contribute to cell survival in some breast cancer cell lines. These results establish *PRNP *as a novel clinically relevant ER stress-regulated gene that may be implicated in increased survival of breast cancer tumor cells. Therefore, targeting the ER stress response and PrP may help in the treatment of a category of breast cancer tumors that are resistant to conventional treatments.

## Abbreviations

4-PBA: 4-Phenyl-Butyric Acid; ΔATF6α: Cleaved activating transcription factor 6α; AARE: Amino-acid-regulatory element; AMV: Avian myeloblastosis virus; ATF4: Activating transcription factor 4; ATF6α: Activating transcription factor 6α; BFA: Brefeldin A; BiP: Immunoglobulin heavy chain binding protein; ChIP: Chromatin immunoprecipitation; CHOP: CCAAT/enhancer-binding protein homologous protein; CHX: Cycloheximide; DMSO: Dimethyl sulfoxide; EDTA: Ethylenediaminetetraacetic acid; eIF2α: Eukaryotic initiation factor 2α; ER: Endoplasmic reticulum; ERS: Estrogen; ERSE: ER stress responsive element; FBS: Fetal bovin serum; FLICA: SR Fluorochrome-labeled inhibitors of caspases; HEK 293: Human embryonic kidney 293; HRP: Horseradish peroxidase; IRE1α: Inositol-requiring enzyme 1α; KD: Knock down; PCR: Polymerase chain reaction; PERK: double-stranded RNA-activated protein kinase-like ER kinase; PR: Progesterone; PrP: Prion protein; *PRNP*: Human prion protein gene; RLU: Relative luminescence units; SERCA: ER Ca-ATPase family; Thps: Thapsigargin; TM: Tunicamycin; TMA: Tissue microarray; TNF: tumor necrosis factor; TRAIL: Tumor Necrosis Factor-Alpha-Related Apoptosis-Inducing Ligand; UPR: Unfolded protein response; XBP1: X-Box protein-1.

## Competing interests

The authors declare that they have no competing interests.

## Authors' contributions

JJ and MAD performed all experiments presented in this paper and participated in the writing of the manuscript. JUS performed the bioinformatics analyses on published databases. CF, SH and MB obtained patient consent and ethical approvals, and established the TMA and obtained medical information from patients. AL directed the study and wrote the manuscript. All authors meet the three authorship requirements described by the Journal. All authors approved the final draft of the manuscript for publication.
